# Aqua­bis­[*N*′-(1,3-dithio­lan-2-ylidene)-2-hy­droxy­benzohydrazidato(0.5−)-κ^2^ *N*′,*O*]sodium(I)

**DOI:** 10.1107/S1600536812023239

**Published:** 2012-05-26

**Authors:** Chahra Bouchameni, Chahrazed Beghidja, Adel Beghidja, Mehdi Boutebdja

**Affiliations:** a Unité de Recherche de Chimie de l’Environnement et Moléculaire Structurale (CHEMS), Faculté des Sciences Exactes, Département de Chimie, Université Mentouri, 25000 Constantine, Algeria

## Abstract

The title compound, [Na(C_10_H_9.5_N_2_O_2_S_2_)_2_(H_2_O)], is a mol­ecular sodium complex with *N*′-(1,3-dithio­lan-2-yl)-2-hy­droxy­benzohydrazide ligands with the negative charge spread evenly over both, and a water mol­ecule. The Na^I^ ion coordination is distorted trigonal–bipyramidal, formed by two N and three O atoms, with the Na^I^ ion lying on a twofold rotation axis. Intra­molecular N—H⋯O hydrogen bonds occur. Mol­ecules pack as discrete units and the crystal packing is stabilized by strong O—H⋯O hydrogen bonds, which give rise to chains along [010]; the chains are inter­linked by strong O—H⋯O hydrogen bonds.

## Related literature

For general background to the 2-salicylihydrazono-1,3-dithiol­ane ligand (H_2_ *L*) and its metal complexes, see: Beghidja *et al.* (2005 ▶, 2006 ▶); Bouchameni *et al.* (2011 ▶). For background to dithio­carbaza­tes, see: Wang *et al.* (2002 ▶); Zhou *et al.* (2007 ▶) and for their biological activity, see: Tarafder *et al.* (2000 ▶, 2001 ▶).
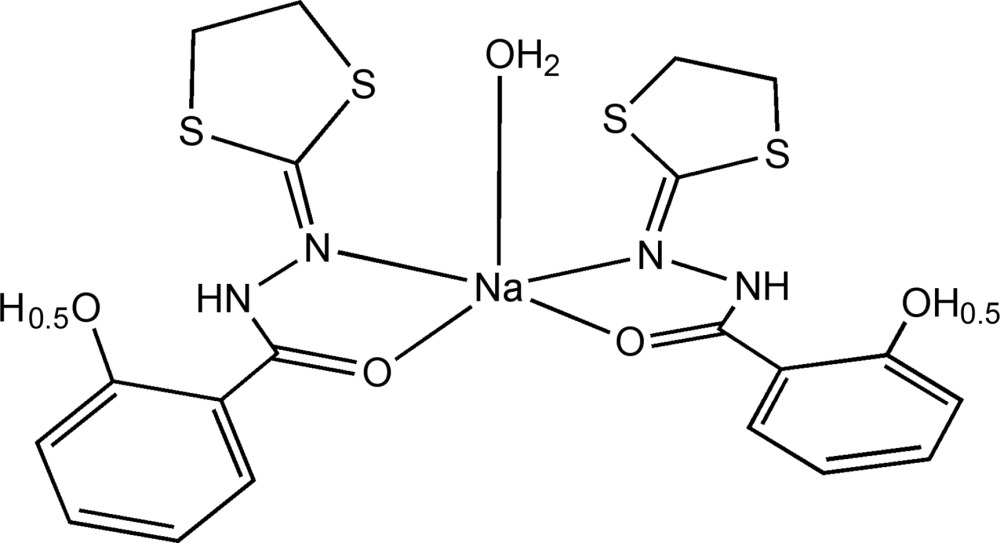


## Experimental

### 

#### Crystal data

[Na(C_10_H_9.5_N_2_O_2_S_2_)_2_(H_2_O)]
*M* *_r_* = 548.68
Monoclinic, 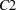

*a* = 16.6960 (16) Å
*b* = 5.9330 (3) Å
*c* = 13.5240 (12) Å
β = 117.804 (3)°
*V* = 1184.99 (17) Å^3^
*Z* = 2
Mo *K*α radiation
μ = 0.46 mm^−1^
*T* = 298 K
0.14 × 0.10 × 0.08 mm

#### Data collection

Nonius KappaCCD diffractometer
3660 measured reflections
2463 independent reflections
2083 reflections with *I* > 2σ(*I*)
*R*_int_ = 0.050

#### Refinement

*R*[*F* ^2^ > 2σ(*F* ^2^)] = 0.047
*wR*(*F* ^2^) = 0.126
*S* = 1.00
2463 reflections
161 parameters
5 restraints
H atoms treated by a mixture of independent and constrained refinement
Δρ_max_ = 0.33 e Å^−3^
Δρ_min_ = −0.43 e Å^−3^
Absolute structure: Flack (1983 ▶), 970 Friedel pairs
Flack parameter: −0.06 (13)

### 

Data collection: *COLLECT* (Nonius, 1998 ▶); cell refinement: *SCALEPACK* (Otwinowski & Minor, 1997 ▶); data reduction: *DENZO* (Otwinowski & Minor, 1997 ▶); program(s) used to solve structure: *SHELXS97* (Sheldrick, 2008 ▶); program(s) used to refine structure: *SHELXL97* (Sheldrick, 2008 ▶); molecular graphics: *PLATON* (Spek, 2009 ▶); software used to prepare material for publication: *SHELXL97*.

## Supplementary Material

Crystal structure: contains datablock(s) global, I. DOI: 10.1107/S1600536812023239/bq2359sup1.cif

Structure factors: contains datablock(s) I. DOI: 10.1107/S1600536812023239/bq2359Isup2.hkl

Additional supplementary materials:  crystallographic information; 3D view; checkCIF report

## Figures and Tables

**Table 1:** Selected bond lengths (Å)

Na1—O1	2.320 (3)
Na1—O3	2.214 (6)
Na1—N2	2.666 (4)

**Table 2:** Hydrogen-bond geometry (Å, °)

*D*—H⋯*A*	*D*—H	H⋯*A*	*D*⋯*A*	*D*—H⋯*A*
N1—H1⋯O2	0.88	1.88	2.596 (4)	137
O2—H2⋯O2^i^	0.89 (5)	1.61 (9)	2.467 (4)	160 (14)
O3—H3⋯O1^ii^	0.95 (4)	1.90 (4)	2.788 (5)	155 (4)

Symmetry codes: (i) 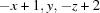; (ii) 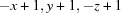.

**Fig. 1.**
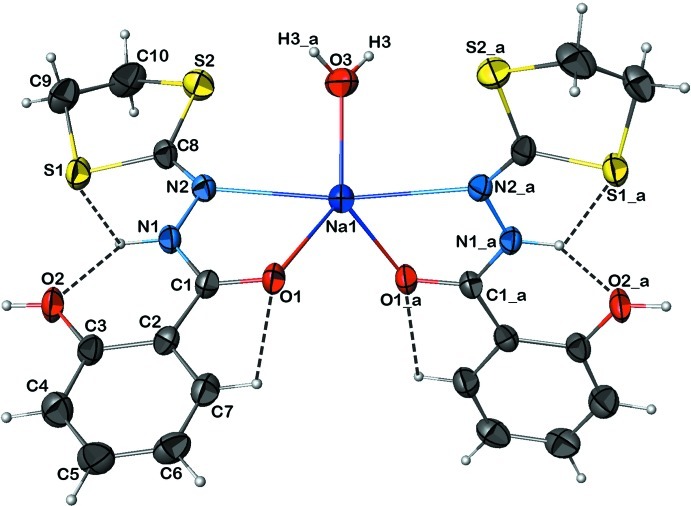
ORTEP view of the title compound with the atom numbering scheme. Displacement ellipsoids for non-H atoms are drawn at the 50% probability level. The intramolecular hydrogen bond is shown as a dashed line. Symmetry code: (a) -x+1, y, -z+1.

**Fig. 2.**
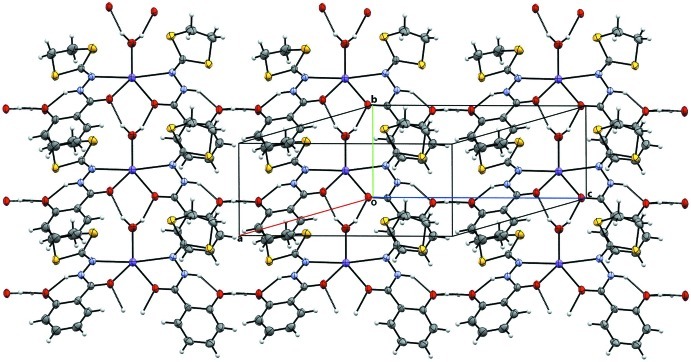
Mercury view of the crystal structure of title compound along the a axis showing the two-dimensional network via a strong hydrogen bonds through O—H···O atoms.

## supplementary crystallographic information

### Comment

Dithiocarbazate R—NH_2_NHCS_2_ (DTC) and its substituted compounds remain of interest to researchers because of their wide variation in structure and properties (Zhou *et al.*, 2007; Wang *et al.*, 2002). Some of these compounds have been widely studies for their antibacterial and antifungal activities (Tarafder *et al.*, 2000; Tarafder *et al.*, 2001). The ligand H_2_L was synthesized as previously described (Beghidja *et al.*, 2005). The molecular structure of the title compound is illustrated in Fig. 1. The sodium cation is chelated by two H_1.5_*L*^-1/2^ bidentate anions coordinated by the hydrazide group *via* O1 and N2 atoms. Na1—O1 = 2.316 (3) Å, Na1—N2 = 2.664 (3) Å. The geometry around the metal is a distorted trigonal bipyramid with a water molecule lying in the axial position through O3. Na1—O3 = 2.213 (5) Å. The crystal structure can be described as a one-dimensional set of chains, interlinked by strong hydrogen-bonds type O—H···O, along the *b* axis, between O3—H3···O1. These chains are interconnected along the *c* axis *via* strong hydrogen-bonds type O—H···O between O2—H2···O2 (Fig 2).

### Experimental

The reaction of H_2_L (0.05 g, 2 *x* 10 ^-4^ mol) with Mn(OH)_3_ (0.0106 g, 10 ^-4^ mol) and NaOH (0.01 g, 2 *x* 10 ^-4^ mol) in ethanol solution leads to a yellow solution which was left to stand undisturbed at room temperature. Several days later, x-ray quality colorless single crystals were obtained by slow evaporation, which were filtrated and characterized.

### Refinement

The positions of the H atoms of the water molecule and hydroxyl group were located in the electron density maps; other H atoms were placed in calculated positions and refined as riding with C—H = 0.95 Å, U_iso_ = 1.2U_eq_(C) for aromatic groups; N—H = 0.86 Å, U_iso_ = 1.2U_eq_(N); O—H = 0.90-0.95 Å, U_iso_ = 1.5U_eq_(O).

### Crystal data

**Table:**

[Na(C_10_H_9.5_N_2_O_2_S_2_)_2_(H_2_O)]	*F*(000) = 568
*M**_r_* = 548.68	*D*_x_ = 1.538 Mg m^−^^3^
Monoclinic, *C*2	Mo *K*α radiation, λ = 0.71073 Å
Hall symbol: C 2y	Cell parameters from 2559 reflections
*a* = 16.6960 (16) Å	θ = 1.0–27.5°
*b* = 5.9330 (3) Å	µ = 0.46 mm^−^^1^
*c* = 13.5240 (12) Å	*T* = 298 K
β = 117.804 (3)°	Plates, colorless
*V* = 1184.99 (17) Å^3^	0.14 × 0.10 × 0.08 mm
*Z* = 2	

### Data collection

**Table:**

Nonius KappaCCD diffractometer	2083 reflections with *I* > 2σ(*I*)
Radiation source: Rotating Anode	*R*_int_ = 0.050
Horizonally mounted graphite crystal monochromator	θ_max_ = 27.5°, θ_min_ = 1.7°
Detector resolution: 9 pixels mm^-1^	*h* = −21→21
φ scans, and ω scans with κ offsets	*k* = −6→7
3660 measured reflections	*l* = −13→17
2463 independent reflections	

### Refinement

**Table:**

Refinement on *F*^2^	Secondary atom site location: difference Fourier map
Least-squares matrix: full	Hydrogen site location: inferred from neighbouring sites
*R*[*F*^2^ > 2σ(*F*^2^)] = 0.047	H atoms treated by a mixture of independent and constrained refinement
*wR*(*F*^2^) = 0.126	*w* = 1/[σ^2^(*F*_o_^2^) + (0.0458*P*)^2^ + 2.9944*P*] where *P* = (*F*_o_^2^ + 2*F*_c_^2^)/3
*S* = 1.00	(Δ/σ)_max_ = 0.021
2463 reflections	Δρ_max_ = 0.33 e Å^−^^3^
161 parameters	Δρ_min_ = −0.43 e Å^−^^3^
5 restraints	Absolute structure: Flack (1983), 970 Friedel pairs
Primary atom site location: structure-invariant direct methods	Flack parameter: −0.06 (13)

### Special details

**Table:**

Geometry. Bond distances, angles etc. have been calculated using the rounded fractional coordinates. All su's are estimated from the variances of the (full) variance-covariance matrix. The cell esds are taken into account in the estimation of distances, angles and torsion angles
Refinement. Refinement on F^2^ for ALL reflections except those flagged by the user for potential systematic errors. Weighted R-factors wR and all goodnesses of fit S are based on F^2^, conventional R-factors R are based on F, with F set to zero for negative F^2^. The observed criterion of F^2^ > 2sigma(F^2^) is used only for calculating -R-factor-obs etc. and is not relevant to the choice of reflections for refinement. R-factors based on F^2^ are statistically about twice as large as those based on F, and R-factors based on ALL data will be even larger.

### Fractional atomic coordinates and isotropic or equivalent isotropic displacement parameters (Å^2^)

**Table:**

	*x*	*y*	*z*	*U*_iso_*/*U*_eq_	Occ. (<1)
S1	0.34168 (7)	0.79324 (19)	0.76027 (8)	0.0330 (3)	
S2	0.33261 (8)	1.0904 (2)	0.57897 (10)	0.0388 (4)	
Na1	0.50000	0.7068 (4)	0.50000	0.0324 (8)	
O1	0.5618 (2)	0.4475 (5)	0.6448 (2)	0.0318 (9)	
O2	0.5000 (2)	0.3636 (5)	0.9088 (2)	0.0348 (10)	
O3	0.50000	1.0799 (9)	0.50000	0.057 (2)	
N1	0.4834 (2)	0.5736 (6)	0.7318 (2)	0.0245 (10)	
N2	0.4434 (2)	0.7412 (6)	0.6540 (3)	0.0261 (10)	
C1	0.5421 (3)	0.4301 (7)	0.7235 (3)	0.0261 (12)	
C2	0.5809 (3)	0.2538 (7)	0.8092 (3)	0.0249 (11)	
C3	0.5575 (3)	0.2237 (7)	0.8968 (3)	0.0282 (11)	
C4	0.5962 (3)	0.0414 (8)	0.9695 (4)	0.0393 (16)	
C5	0.6562 (3)	−0.1025 (9)	0.9594 (4)	0.0400 (16)	
C6	0.6809 (3)	−0.0701 (8)	0.8755 (4)	0.0373 (14)	
C7	0.6425 (3)	0.1041 (8)	0.8012 (3)	0.0325 (12)	
C8	0.3821 (3)	0.8554 (7)	0.6639 (3)	0.0262 (11)	
C9	0.2806 (3)	1.0562 (9)	0.7390 (4)	0.0400 (16)	
C10	0.2425 (3)	1.1176 (9)	0.6176 (4)	0.0453 (16)	
H1	0.47040	0.56020	0.78750	0.0290*	
H2	0.512 (8)	0.379 (19)	0.980 (3)	0.0680*	0.500
H3	0.470 (4)	1.171 (8)	0.435 (3)	0.0680*	
H4	0.58030	0.01670	1.02760	0.0470*	
H5	0.68110	−0.22480	1.01000	0.0480*	
H6	0.72380	−0.16700	0.86960	0.0450*	
H7	0.65820	0.12360	0.74260	0.0390*	
H9A	0.23110	1.03850	0.75920	0.0480*	
H9B	0.32190	1.17610	0.78640	0.0480*	
H10A	0.21950	1.27430	0.60530	0.0540*	
H10B	0.19180	1.01580	0.57130	0.0540*	

### Atomic displacement parameters (Å^2^)

**Table:**

	*U*^11^	*U*^22^	*U*^33^	*U*^12^	*U*^13^	*U*^23^
S1	0.0373 (6)	0.0381 (6)	0.0316 (5)	0.0081 (5)	0.0228 (5)	0.0031 (5)
S2	0.0465 (7)	0.0349 (6)	0.0353 (6)	0.0119 (5)	0.0194 (5)	0.0097 (5)
Na1	0.0457 (14)	0.0278 (13)	0.0329 (12)	0.0000	0.0260 (11)	0.0000
O1	0.0435 (17)	0.0330 (17)	0.0300 (15)	0.0067 (13)	0.0265 (14)	0.0027 (12)
O2	0.0449 (18)	0.0407 (19)	0.0274 (15)	0.0150 (15)	0.0240 (15)	0.0072 (13)
O3	0.113 (5)	0.029 (3)	0.040 (3)	0.0000	0.044 (3)	0.0000
N1	0.0286 (17)	0.0287 (18)	0.0195 (15)	0.0012 (15)	0.0139 (13)	0.0016 (14)
N2	0.0321 (18)	0.0259 (19)	0.0225 (16)	0.0035 (15)	0.0145 (14)	0.0029 (12)
C1	0.030 (2)	0.028 (2)	0.025 (2)	−0.0002 (17)	0.0167 (17)	−0.0024 (16)
C2	0.0249 (18)	0.027 (2)	0.0217 (17)	−0.0008 (16)	0.0100 (14)	−0.0048 (15)
C3	0.030 (2)	0.033 (2)	0.0213 (18)	0.0045 (17)	0.0116 (16)	0.0001 (16)
C4	0.046 (3)	0.041 (3)	0.029 (2)	0.008 (2)	0.016 (2)	0.0058 (18)
C5	0.045 (3)	0.036 (3)	0.029 (2)	0.012 (2)	0.009 (2)	0.0066 (19)
C6	0.036 (2)	0.033 (3)	0.037 (2)	0.011 (2)	0.012 (2)	−0.002 (2)
C7	0.033 (2)	0.036 (2)	0.033 (2)	−0.001 (2)	0.0193 (18)	−0.005 (2)
C8	0.029 (2)	0.030 (2)	0.0190 (18)	−0.0005 (16)	0.0108 (16)	0.0010 (15)
C9	0.038 (2)	0.044 (3)	0.043 (3)	0.009 (2)	0.023 (2)	−0.003 (2)
C10	0.040 (2)	0.045 (3)	0.045 (3)	0.022 (2)	0.015 (2)	0.003 (2)

### Geometric parameters (Å, º)

**Table:**

S1—C8	1.764 (5)	N1—H1	0.8800
S1—C9	1.812 (6)	C1—C2	1.469 (6)
S2—C8	1.750 (4)	C2—C3	1.421 (6)
S2—C10	1.815 (6)	C2—C7	1.401 (7)
Na1—O1	2.320 (3)	C3—C4	1.401 (6)
Na1—O3	2.214 (6)	C4—C5	1.371 (8)
Na1—N2	2.666 (4)	C5—C6	1.390 (7)
Na1—O1^i^	2.320 (3)	C6—C7	1.374 (6)
Na1—N2^i^	2.666 (4)	C9—C10	1.503 (7)
O1—C1	1.256 (5)	C4—H4	0.9500
O2—C3	1.335 (6)	C5—H5	0.9500
O2—H2	0.89 (5)	C6—H6	0.9500
O3—H3^i^	0.95 (4)	C7—H7	0.9500
O3—H3	0.95 (4)	C9—H9A	0.9900
N1—N2	1.373 (5)	C9—H9B	0.9900
N1—C1	1.342 (6)	C10—H10A	0.9900
N2—C8	1.285 (6)	C10—H10B	0.9900
			
S1···N1	2.880 (4)	C3···H1	2.5100
S1···C4^ii^	3.614 (5)	C3···H2^ix^	2.60 (11)
S2···C10^iii^	3.660 (5)	C4···H9B^xii^	3.0300
S2···O3	3.4263 (15)	C4···H2^ix^	2.98 (12)
S1···H1	2.4300	C4···H4^ix^	2.9700
S1···H7^iv^	3.1300	C6···H5^xiii^	2.9300
S1···H4^ii^	2.8600	C7···H5^xiii^	3.0300
S2···H10A^iii^	2.9100	C7···H3^viii^	2.88 (4)
S2···H10B^v^	3.1500	C9···H6^xiv^	2.8800
Na1···C10^vi^	3.633 (6)	C9···H4^ii^	2.9300
Na1···C10^iii^	3.633 (6)	H1···S1	2.4300
Na1···H10B^vi^	3.1000	H1···O2	1.8800
Na1···H10B^iii^	3.1000	H1···C3	2.5100
O1···O3^vii^	2.788 (5)	H1···H2	2.5900
O1···N2	2.681 (5)	H2···H1	2.5900
O1···C9^vi^	3.323 (7)	H2···H4	2.3800
O1···O3^viii^	2.788 (5)	H2···O2^ix^	1.61 (9)
O1···C10^vi^	3.360 (7)	H2···C3^ix^	2.60 (11)
O2···C4^ix^	3.378 (6)	H2···C4^ix^	2.98 (12)
O2···N1	2.596 (4)	H3···O1^xi^	1.90 (4)
O2···C3^ix^	3.298 (5)	H3···C1^xi^	2.57 (4)
O2···O2^ix^	2.467 (4)	H3···C2^xi^	3.04 (4)
O3···O1^x^	2.788 (5)	H3···C7^xi^	2.88 (4)
O3···S2^i^	3.4263 (15)	H3···H7^xi^	2.3700
O3···O1^xi^	2.788 (5)	H4···H2	2.3800
O3···S2	3.4263 (15)	H4···S1^xii^	2.8600
O1···H7	2.4600	H4···O2^ix^	2.8000
O1···H10B^vi^	2.8100	H4···C4^ix^	2.9700
O1···H3^viii^	1.90 (4)	H4···C9^xii^	2.9300
O1···H9A^vi^	2.5700	H4···H4^ix^	2.4200
O2···H2^ix^	1.61 (8)	H4···H9B^xii^	2.4600
O2···H9B^vii^	2.8700	H5···C6^xv^	2.9300
O2···H4^ix^	2.8000	H5···C7^xv^	3.0300
O2···H1	1.8800	H6···C9^xvi^	2.8800
N1···S1	2.880 (4)	H6···H9A^xvi^	2.3400
N1···O1	2.257 (5)	H6···H9B^xvi^	2.5600
N1···O2	2.596 (4)	H7···O1	2.4600
N2···O1	2.681 (5)	H7···H3^viii^	2.3700
C3···O2^ix^	3.298 (5)	H7···S1^vi^	3.1300
C4···O2^ix^	3.378 (6)	H9A···O1^iv^	2.5700
C4···S1^xii^	3.614 (5)	H9A···C1^iv^	3.0300
C4···C9^xii^	3.495 (7)	H9A···H6^xiv^	2.3400
C9···O1^iv^	3.323 (7)	H9B···O2^x^	2.8700
C9···C4^ii^	3.495 (7)	H9B···C4^ii^	3.0300
C10···O1^iv^	3.360 (7)	H9B···H4^ii^	2.4600
C10···Na1^iv^	3.633 (6)	H9B···H6^xiv^	2.5600
C10···Na1^v^	3.633 (6)	H10A···S2^v^	2.9100
C10···S2^v^	3.660 (5)	H10B···Na1^iv^	3.1000
C1···H9A^vi^	3.0300	H10B···O1^iv^	2.8100
C1···H3^viii^	2.57 (4)	H10B···S2^iii^	3.1500
C2···H3^viii^	3.04 (4)	H10B···Na1^v^	3.1000
			
C8—S1—C9	94.8 (2)	O2—C3—C2	121.1 (4)
C8—S2—C10	94.8 (2)	O2—C3—C4	121.2 (4)
O1—Na1—O3	131.54 (8)	C3—C4—C5	122.0 (5)
O1—Na1—N2	64.63 (11)	C4—C5—C6	120.3 (5)
O1—Na1—O1^i^	96.91 (13)	C5—C6—C7	119.1 (5)
O1—Na1—N2^i^	122.01 (13)	C2—C7—C6	122.0 (4)
O3—Na1—N2	85.61 (9)	S1—C8—S2	115.2 (3)
O1^i^—Na1—O3	131.54 (8)	S1—C8—N2	124.4 (3)
O3—Na1—N2^i^	85.61 (9)	S2—C8—N2	120.5 (3)
O1^i^—Na1—N2	122.01 (13)	S1—C9—C10	107.6 (4)
N2—Na1—N2^i^	171.22 (15)	S2—C10—C9	107.9 (4)
O1^i^—Na1—N2^i^	64.63 (11)	C3—C4—H4	119.00
Na1—O1—C1	125.5 (3)	C5—C4—H4	119.00
C3—O2—H2	113 (8)	C4—C5—H5	120.00
Na1—O3—H3	125 (3)	C6—C5—H5	120.00
Na1—O3—H3^i^	125 (3)	C5—C6—H6	120.00
H3—O3—H3^i^	111 (4)	C7—C6—H6	120.00
N2—N1—C1	120.6 (3)	C2—C7—H7	119.00
Na1—N2—N1	108.7 (2)	C6—C7—H7	119.00
Na1—N2—C8	135.3 (3)	S1—C9—H9A	110.00
N1—N2—C8	115.4 (4)	S1—C9—H9B	110.00
C1—N1—H1	120.00	C10—C9—H9A	110.00
N2—N1—H1	120.00	C10—C9—H9B	110.00
N1—C1—C2	117.2 (4)	H9A—C9—H9B	108.00
O1—C1—N1	120.6 (4)	S2—C10—H10A	110.00
O1—C1—C2	122.3 (4)	S2—C10—H10B	110.00
C1—C2—C7	117.4 (4)	C9—C10—H10A	110.00
C3—C2—C7	118.8 (4)	C9—C10—H10B	110.00
C1—C2—C3	123.8 (4)	H10A—C10—H10B	108.00
C2—C3—C4	117.8 (4)		
			
C9—S1—C8—S2	−11.2 (3)	N2—N1—C1—C2	−179.2 (4)
C9—S1—C8—N2	168.7 (4)	Na1—N2—C8—S1	163.7 (2)
C8—S1—C9—C10	35.8 (4)	Na1—N2—C8—S2	−16.4 (6)
C10—S2—C8—S1	−11.6 (3)	N1—N2—C8—S1	−5.8 (5)
C10—S2—C8—N2	168.5 (4)	N1—N2—C8—S2	174.1 (3)
C8—S2—C10—C9	36.2 (4)	O1—C1—C2—C3	−177.5 (4)
O3—Na1—O1—C1	60.2 (4)	O1—C1—C2—C7	0.7 (6)
N2—Na1—O1—C1	2.4 (3)	N1—C1—C2—C3	2.1 (6)
O1^i^—Na1—O1—C1	−119.8 (3)	N1—C1—C2—C7	−179.8 (4)
N2^i^—Na1—O1—C1	175.9 (3)	C1—C2—C3—O2	−2.7 (7)
O1—Na1—N2—N1	−1.8 (2)	C1—C2—C3—C4	176.8 (4)
O1—Na1—N2—C8	−171.8 (5)	C7—C2—C3—O2	179.1 (4)
O3—Na1—N2—N1	−142.4 (2)	C7—C2—C3—C4	−1.4 (6)
O3—Na1—N2—C8	47.6 (4)	C1—C2—C7—C6	−178.4 (4)
O1^i^—Na1—N2—N1	80.2 (3)	C3—C2—C7—C6	−0.1 (7)
O1^i^—Na1—N2—C8	−89.8 (4)	O2—C3—C4—C5	−179.2 (4)
Na1—O1—C1—N1	−2.7 (6)	C2—C3—C4—C5	1.3 (7)
Na1—O1—C1—C2	176.9 (3)	C3—C4—C5—C6	0.3 (8)
C1—N1—N2—Na1	1.5 (4)	C4—C5—C6—C7	−1.8 (8)
C1—N1—N2—C8	173.7 (4)	C5—C6—C7—C2	1.7 (7)
N2—N1—C1—O1	0.4 (6)	S1—C9—C10—S2	−48.2 (4)

Symmetry codes: (i) −*x*+1, *y*, −*z*+1; (ii) −*x*+1, *y*+1, −*z*+2; (iii) −*x*+1/2, *y*−1/2, −*z*+1; (iv) *x*−1/2, *y*+1/2, *z*; (v) −*x*+1/2, *y*+1/2, −*z*+1; (vi) *x*+1/2, *y*−1/2, *z*; (vii) *x*, *y*−1, *z*; (viii) −*x*+1, *y*−1, −*z*+1; (ix) −*x*+1, *y*, −*z*+2; (x) *x*, *y*+1, *z*; (xi) −*x*+1, *y*+1, −*z*+1; (xii) −*x*+1, *y*−1, −*z*+2; (xiii) −*x*+3/2, *y*+1/2, −*z*+2; (xiv) *x*−1/2, *y*+3/2, *z*; (xv) −*x*+3/2, *y*−1/2, −*z*+2; (xvi) *x*+1/2, *y*−3/2, *z*.

### Hydrogen-bond geometry (Å, º)

**Table:**

*D*—H···*A*	*D*—H	H···*A*	*D*···*A*	*D*—H···*A*
N1—H1···S1	0.8800	2.4300	2.880 (4)	112.00
N1—H1···O2	0.8800	1.8800	2.596 (4)	137.00
O2—H2···O2^ix^	0.89 (5)	1.61 (9)	2.467 (4)	160 (14)
O3—H3···O1^xi^	0.95 (4)	1.90 (4)	2.788 (5)	155 (4)
C4—H4···S1^xii^	0.95	2.86	3.614 (5)	137
C7—H7···O1	0.95	2.46	2.790 (5)	100
C9—H9*A*···O1^iv^	0.99	2.57	3.323 (7)	133

Symmetry codes: (iv) *x*−1/2, *y*+1/2, *z*; (ix) −*x*+1, *y*, −*z*+2; (xi) −*x*+1, *y*+1, −*z*+1; (xii) −*x*+1, *y*−1, −*z*+2.
